# Outcomes of Tympanic Membrane Regenerative Surgery Using Gelatin Sponge, Recombinant Basic Fibroblast Growth Factor, and Fibrin Glue

**DOI:** 10.7759/cureus.75259

**Published:** 2024-12-07

**Authors:** Hiroshi Hyakusoku, Jun Aoyama, Toshimasa Aoki, Risa Kamoshida, Meijin Nakayama

**Affiliations:** 1 Otolaryngology, Yokosuka Kyosai Hospital, Yokosuka, JPN

**Keywords:** basic fibroblast growth factor, fibrin glue, gelatin sponges, regenerative treatment, tympanic membrane perforation

## Abstract

Objective

We evaluated the outcomes of tympanic membrane regenerative treatment using gelatin sponge, recombinant basic fibroblast growth factor (bFGF), and fibrin glue at Yokosuka Kyosai Hospital.

Methodology

We enrolled a total of 42 patients with tympanic membrane perforations (TMPs) (44 ears; right:left = 21:23) that were treated using gelatin sponge, recombinant bFGF, and fibrin glue between July 2020 and December 2023 at Yokosuka Kyosai Hospital. TMP closure rates, improvement of hearing level, and complications were retrospectively included in the evaluation items. TMP was evaluated at least one month after surgery. The treatment was repeated up to four times until the TMP was completely closed.

Results

The perforation size distribution was as follows: grade I, 30 ears (68.1%); grade II, 11 ears (25.0%); and grade III, three ears (6.8%). The overall closure rate for TMP was 84.1% (37/44). Closure was achieved in 72.7% (32/44) after the first treatment and 11.4% (5/44) after the second treatment. No closures were achieved after the third and fourth treatments. Factors contributing to the incomplete closure of TMPs included patient refusal of surgery more than once, and the disappearance of the gelatin sponge one week after each surgery. Mean air-conduction thresholds and mean air-bone gaps improved after TMP closure in successful patients; however, no change in mean bone-conduction thresholds was observed at any frequency. No serious complications were observed.

Conclusions

We found high success rates for TMP closure, good hearing recovery, and no severe complications. Our findings suggest that our novel technique has favorable outcomes.

## Introduction

Tympanic membrane perforation (TMP) can cause hearing loss, ear fullness, tinnitus, and sometimes infection, resulting in otorrhea. These conditions often interfere with daily life. Furthermore, using a hearing aid with a perforated tympanic membrane (TM) hinders effective sound transmission and increases the risk of infection, as the ear canal becomes a closed space. However, closing the TM can alleviate or reduce these symptoms.

TMP is closed by surgical procedures such as myringoplasty or tympanoplasty [1-3]. Conventional TM reconstruction surgeries typically require harvesting autologous tissue as a scaffold, which can result in a residual air-bone gap due to the thickness of the graft compared to the native TM. Additionally, tissue extraction is invasive and associated with higher medical costs. Therefore, a simpler and more cost-effective surgical technique is necessary.

In 2011, Kanemaru et al. reported that after removing the edge of the perforation, TMP could be easily closed by inserting a gelatin sponge immersed in basic fibroblast growth factor (bFGF) into the perforation, followed by dripping fibrin glue over the sponge to seal the surgical site [4]. This surgery eliminates the need for harvesting autologous tissue, allowing for the regeneration of the TM to closely resemble its natural structure. In this procedure, a gelatin sponge soaked in bFGF serves as a scaffold, replacing the need for autologous tissue and enhancing the TM's intrinsic self-renewal capacity.

In 2018, a kit called RytympaTM (Nobelpharma Co. Ltd., Tokyo, Japan), which consisted of gelatin sponges and recombinant bFGF, received pharmaceutical approval in Japan. Thereafter, the technique gained prevalence and is currently being used in clinical practice in Japan. In the facility where this surgical procedure was developed, a closure rate of over 90% was reported [5]. Herein, we aimed to evaluate the outcomes of TMP closure surgeries using gelatin sponge, recombinant bFGF, and fibrin glue in our department.

## Materials and methods

Cases

We included patients who underwent TMP closure surgeries using gelatin sponge, recombinant bFGF, and fibrin glue between July 2020 and December 2023 at Yokosuka Kyosai Hospital.

Treatments

Multiple surgeons performed. For ears with observed effusion before surgery, treatment was administered until the middle ear became dry before proceeding with the surgery. TMP closure surgeries were performed as previously described [5]. Briefly, after confirming that local anesthesia induced by 4% lidocaine was effective, the edge of the TMP was surgically disrupted and removed. A gelatin sponge immersed in bFGF was then inserted into the perforation, followed by dripping fibrin glue over the sponge to seal the surgical site. These procedures were performed under a microscope. However, for four cases in which patients refused to undergo surgery under local anesthesia, surgery was performed under general anesthesia, using an endoscope. After surgery, patients were instructed not to put water or any other substances into their ears and avoid activities that could cause pressure changes in their ears such as blowing their nose or sniffing. Therefore, we scheduled surgeries to avoid patients' hay fever seasons and instructed those regularly taking anti-allergic medications to continue their use. The TMP was evaluated more than one month after surgery. If the TMP persisted, the same procedure was repeated up to four times.

Outcomes

The rate of complete TMP closure was estimated through a review of medical charts considering factors such as the size of the TMP, the number of surgical procedures, and the patient’s history of TM surgeries. The size of TMP was classified into three grades: grade I (TMP < 1/3 of the entire TM), grade II (TMP between 1/3 and 2/3 of the entire TM), and grade III (TMP > 2/3 of the entire TM). The hearing level of each frequency, pure tone audiometry (PTA; average of 500, 1,000, 2,000, and 3,000 Hz), and bone-conduction thresholds and air-bone gaps (average of 250, 500, 1,000, 2,000, and 4,000 Hz) were evaluated before surgery and more than one month after closure.

Statistical analysis

The averages of air-conduction thresholds, PTA, air-bone gaps, and bone-conduction thresholds before surgery and after TMP closure were compared using a Wilcoxon matched-pairs signed rank test. For all comparisons, *P* < 0.05 was defined as significant.

## Results

General characteristics

A total of 44 TMs were enrolled in this study. The cohort consisted of 21 males and 21 females with a median age of 70.5 years (range 8-87). A total of 21 right ears and 23 left ears were included. Additionally, one male and one female had perforations in both TMs.

TM condition

The size of TMPs before surgery was grade I in 30 TMs (68.2%), grade II in 11 TMs (25.0%), and grade III in three TMs (6.8%). Three patients had undergone one myringoplasty and experienced failure. The remaining 41 TMs were fresh TMPs.

TMP closure

The overall TMP closure rate was 84.1% (37/44) (Table 1). Complete TMP closure was achieved in 72.7% (32/44) after the first surgery. Three patients with incomplete TMP closure after the first surgery refused further surgery. Complete TMP closure was achieved in an additional 11.4% (5/44) of cases following a second surgery, performed on nine patients. One patient with incomplete TMP closure after the second surgery refused the third surgery. In two patients, the gelatin sponge had disappeared one week after both the first and second surgeries, leading to the cessation of further surgeries. One patient underwent four surgeries without TM closure. In this case, the gelatin sponge disappeared one week after each surgery.

**Table 1:** Outcomes for the number of treatments required to close a tympanic membrane (TM) and grade of TMP. Three patients with incomplete tympanic membrane perforation (TMP) closure after the first surgery declined additional surgery. One patient with incomplete TMP closure after the second surgery refused further surgery. In two patients, the gelatin sponge had disappeared one week after both the first and second surgeries, resulting in the discontinuation of subsequent surgeries.

Number of treatments	Size of perforation			
	Grade I (*n* = 30)	Grade II (*n* = 11)	Grade III (*n* = 3)	Total (*n* = 45)
One treatment	23/30	8/11	1/3	32/44 (72.7%)
Second treatment	3/30 (4 surgeries; 3 quitted)	2/11 (3 surgeries)	0/3 (2 surgeries)	5/44 (11.4%) (3 quitted)
Third treatment	0/30 (no surgeries; 1 quitted)	0/11 (no surgeries; 1 quitted)	0/3 (1 surgery; 1 quitted)	0/44 (0.0%) (3 quitted)
Fourth treatment	0/30 (no surgeries)	0/11 (no surgeries)	0/3 (1 surgery)	0/44 (0.0%) (no quitted)
Closure rates	26/30 (86.7%)	10/11 (90.9%)	1/3 (33.3%)	37/44 (84.1%) (6 quitted)

**Figure 1:**
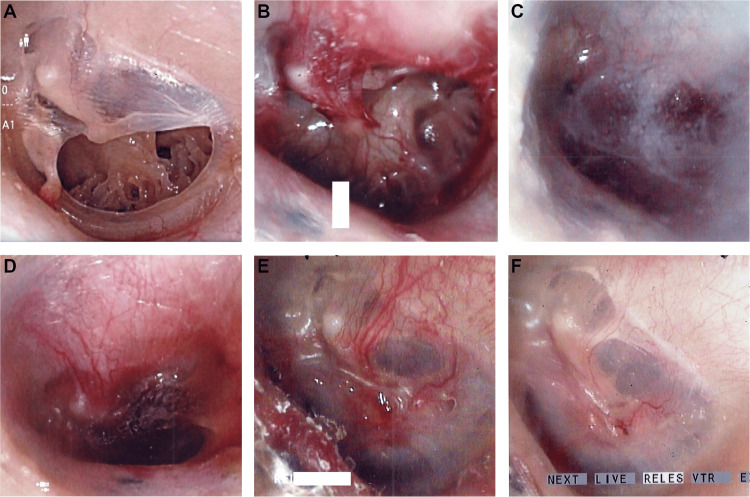
Images from a 30-year-old female with left TMP. (A) Grade II TMP with calcification. (B) The edge of the TMP was mechanically disrupted, including the calcification. (C) A gelatin sponge immersed in bFGF was inserted into the perforation, followed by dripping fibrin glue over the sponge to seal the surgical site. (D) One week after surgery. (E) One and a half months after surgery. The TMP was closed. (F) Two months after surgery. The regenerated TM resembled a natural TM. TMP, tympanic membrane perforation; TM, tympanic membrane

Figure 1 shows a successful closure and regeneration of a TM from the left ear of a 30-year-old female. The patient had chronic otitis media due to recurrent acute otitis media in her childhood. The TMP was completely closed after the first surgery.

Table 2 shows TMs that were not closed. The factors contributing to the incomplete closure of TMPs were patient refusal of surgery more than once and the disappearance of the gelatin sponge one week after each surgery. Specifically, the gelatin sponge had disappeared one week after surgery in a total of 10 TMs (five TMs after the first surgery, three TMs after the second surgery, one TM after the third surgery, and one TM after the fourth surgery). All these TMs were not closed.

**Table 2:** Characteristics of patients for which TMPs were not closed. TMP, tympanic membrane perforation

Age/Sex	Side	TMP size	Factors for closure failure
60/F	L	I	The pinhole remained after the first surgery. The second surgery was refused.
71/M	L	I	The pinhole remained after the first surgery. The second surgery was refused.
73/M	L	III	The gelatin sponge disappeared one week after treatment every time (four times) because of sniffing or ear cleaning.
74/F	L	I	The pinhole remained after the second surgery. The third surgery was refused.
74/M	L	II	The gelatin sponge disappeared one week after both the first and second treatments because of sniffing, leading to the cessation of further treatment.
81/F	R	I	The second surgery was refused.
84/M	L	III	The gelatin sponge disappeared one week after both the first and second treatments because of sniffing, leading to the cessation of further treatment.

Hearing levels

**Figure 2:**
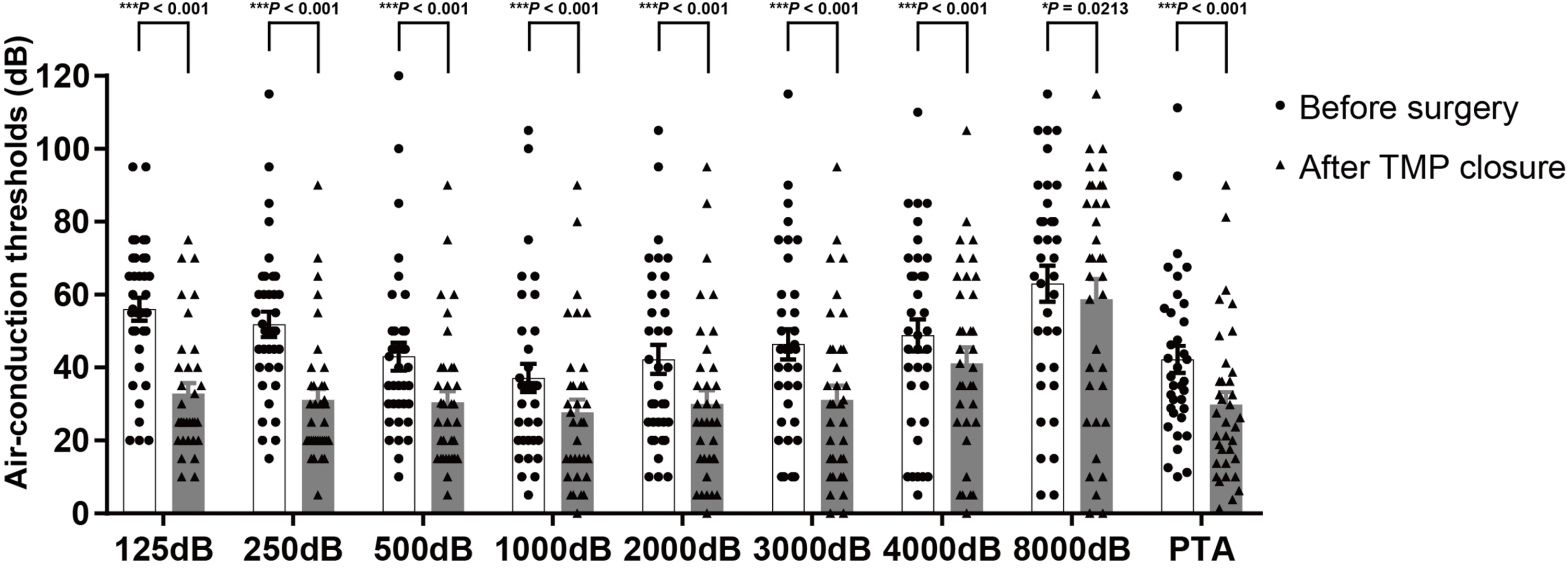
The distribution of air-conduction thresholds and PTA before surgery and after TMP closure. The average PTA was significantly improved from 42.2 ± 3.8 dB before surgery to 29.8 ± 3.6 dB (*P* = 0.0201). All of the mean air-conduction thresholds improved (125 Hz; 56.0 ± 3.3 dB vs. 32.9 ± 2.9 dB, 250Hz; 51.9 ± 3.6 dB vs. 31.1 ± 3.1 dB, 500 Hz; 43.0 ± 3.9 dB vs. 30.4 ± 3.3 dB, 1,000 Hz; 37.1 ± 4.0 dB vs. 27.7 ± 3.6 dB, 2,000 Hz; 42.3 ± 4.1 dB vs. 30.0 ± 3.9 dB, 3,000 Hz; 46.4 ± 4.3 dB vs. 31.1 ± 4.1 dB, 4,000 Hz; 48.9 ± 4.5 dB vs. 41.1 ± 4.5 dB, 8,000 Hz; 63.0 ± 5.1 dB vs. 58.7 ± 5.7 dB). Columns, mean number of dB; bars, SEM. *P < 0.05, ***P < 0.001. TMP, tympanic membrane perforation; PTA, pure tone audiometry; SEM, standard error of the mean

**Figure 3:**
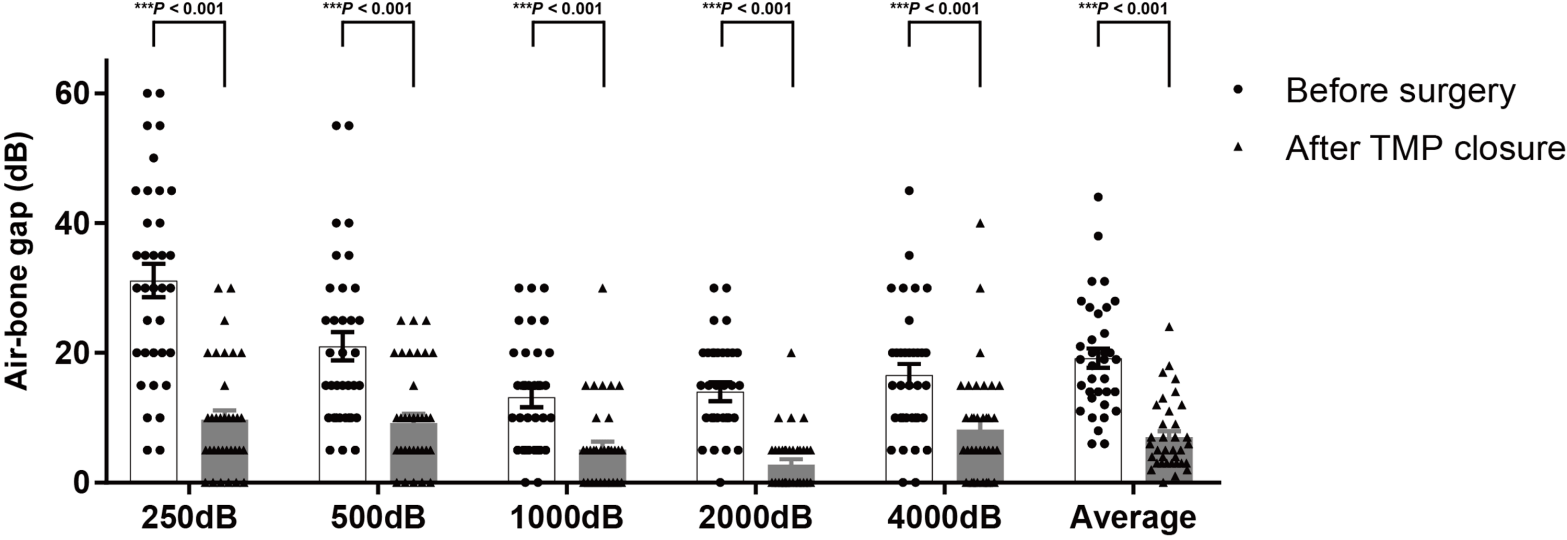
The distribution of air-bone gaps before surgery and after TMP closure. Mean air-bone gaps significantly improved at all frequencies (250 Hz; 31.4 ± 2.6 dB vs. 9.7 ± 1.4 dB, 500 Hz; 21.0 ± 2.2 dB vs. 9.2 ± 1.5 dB, 1,000 Hz; 13.1 ± 1.5 dB vs. 5.1 ± 1.2 dB, 2,000 Hz; 14.0 ± 1.4 dB vs. 2.8 ± 0.8 dB, 4,000 Hz; 16.6 ± 1.7 dB vs. 8.2 ± 1.5 dB). Columns, mean number of dB; bars, SEM. ***P < 0.001. TMP, tympanic membrane perforation; SEM, standard error of the mean

**Figure 4:**
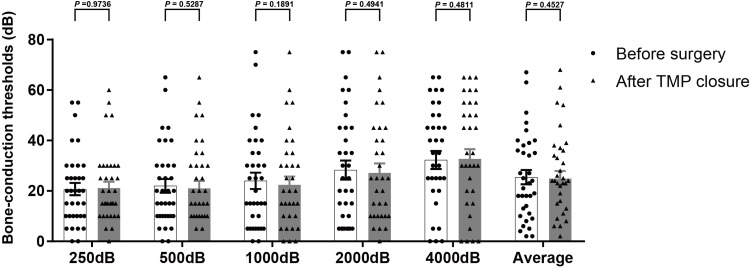
The distribution of each bone-conduction threshold before surgery and after TMP closure. No significant change was observed. TMP, tympanic membrane perforation

Excluding one ear that developed postoperative otitis media with effusion, hearing evaluations were performed in the 35 ears with successful TMP closure. Figure 2 shows the distribution of each air-conduction threshold and PTA before surgery and after TMP closure. Average PTA significantly improved from 42.2 ± 3.8 dB before surgery to 29.8 ± 3.6 dB (*P* < 0.001) after TMP closure in successful patients. The mean air-conduction thresholds significantly improved at all frequencies. The mean air-bone gaps also showed significant improvement across all frequencies, resulting in values within 10 dB at all frequencies after TMP closure (Figure 3). The average reduction in air-bone gaps to 5 dB or less was achieved in 50.0% (18/35) of ears, to less than 10 dB in 72.2% (26/35), and to less than 20 dB in 97.2% (34/35). On the other hand, no significant change was observed (Figure 4).

Complications

Painful infections occurred in two ears, which improved with oral antibiotics, resulting in successful closure. One ear developed otitis media with effusion, resulting in spontaneous perforation. Three patients developed transient vertigo due to local anesthesia. No serious complications were observed.

## Discussion

TMP closure rates

The overall closure rates for complete TMP were lower than in previous reports [4,5]. This was attributed to four patients refusing second or third surgeries, and three patients who had ear cleaning, nose blowing, and sniffing habits, which may have led to the disappearance of the sponges after one week. Previous reports excluded patients who refused second and subsequent surgeries; thus, if these patients were excluded, the closure rate was 97.4% (37/38), which was consistent with previous reports. Our results were consistent with closure rates reported for conventional myringoplasty using autologous tissue [1,6,7]. To reduce dropouts from the second procedure onwards, it is crucial to explain to patients that they may require up to four surgeries before achieving complete closure and that there is a possibility of closure after the second, third, or fourth surgeries even if closure is not achieved during the first surgery. Furthermore, patients who have a habit of ear cleaning or nose sniffing should be instructed to refrain from inserting anything into their ears and should also seek treatment for allergic rhinitis. Surgery should be avoided during the hay fever season.

Hearing levels

All frequencies showed improved air-conduction hearing, and this was prominent in lower frequencies. This was due to larger air-bone gaps for low frequencies, as previously reported by Kanai et al. [5]. On the other hand, there was no improvement in bone-conduction hearing, which is inconsistent with the slight improvement reported by Kanai et al. In vivo, bFGF has been reported to improve hearing in cases of acute sensorineural hearing loss and to have a protective effect on the inner ear [8,9]. However, in this study, there were no cases of acute sensorineural hearing loss. The effect of bFGF on improving bone-conduction hearing remains to be elucidated.

In conventional myringoplasty, the use of autologous graft tissue often results in a thicker TM, which can cause a persistent air-bone gap [10]. Our novel technique regenerates the TM to resemble a natural TM. The regenerated TM is thinner, which may reduce the air-bone gap. In this study, we found smaller air-bone gaps than those reported in previous myringoplasty studies [11,12]. The air-bone gaps were the smallest at 2,000 Hz and within 10 dB on average at all frequencies. Since all patients underwent surgery only after confirming the absence of soft tissue in the middle ear on computed tomography, and because the air-bone gaps were smallest at 2,000 Hz, these findings suggest that some patients had damage or fixation of the ossicular chain due to long-term chronic otitis media or otosclerosis [13,14].

Factors contributing to incomplete closure of TMP

Kanai et al. [5] attributed TMPs that remained unclosed to factors such as postoperative infection; ear irradiation; ear cleaning, nose blowing, and sniffing habits; post-tympanoplasty; and excessive disruption of the edge of the TMP. In this study, closure of TMP was unsuccessful in some cases due to ear cleaning, nose blowing, and sniffing habits. When you blow or sniff your nose, the pressure in your middle ear can increase or decrease by approximately 200 mmH_2_O due to airflow through the Eustachian tube [15,16]. On the other hand, two cases experienced postoperative infections but resulted in successful closure. As the middle ear cavity was treated until it was dry before surgery, and then the surgery was performed, even in cases of postoperative infection, the regeneration process may have been activated, which led to complete closure. In addition, in the event of an infection, it is important to administer oral antibiotics and avoid putting anything in the ear, including ear drops.

All three cases who underwent myringoplasty previously attained closure after the first surgery. This suggests that a previous minor TM surgery does not affect closure.

Complications

There were no severe complications observed in this study. Although two TMs were infected, the patients were administered oral antibiotics, and the TMPs were subsequently closed. However, it is necessary to avoid putting anything into the ear including antibiotics drops during and after surgery due to the risk of infection. One patient who had eustachian tube stenosis had TM closure, but later developed otitis media with effusion, and the TM was re-perforated. Eustachian tube function was assessed preoperatively using sonotubometry in most patients. However, in this patient, the patient’s Eustachian tube function was not evaluated, as sonotubometry was not performed. In cases of Eustachian tube dysfunction, patients were informed of the potential risk of developing otitis media with effusion following TM closure. Despite these risks, patients consented to proceed with surgery. One patient involved an 84-year-old male diagnosed with Eustachian tube dysfunction based on sonotubometry results. Although he underwent two surgical procedures, the gelatin sponge disappeared one week after both treatments because of habitual sniffing, leading to the cessation of further treatment. In contrast, six patients with patulous Eustachian tubes successfully underwent surgical closure of the TM without complications. It may be necessary to consider whether surgery should be performed in cases with poor eustachian tube function as well as in cases undergoing myringoplasty [17,18].

Although some patients experienced temporary vertigo due to local anesthesia, all patients recovered. However, it is important to inform the patient of the possibility of vertigo due to the surgery and to advise them to avoid driving on the day of surgery.

This surgery has the potential to result in epidermal pearl formation, as bFGF can stimulate the proliferation of both connective tissue and epidermal cells [19,20]. Kanai et al. [5] reported a postoperative tympanic cholesteatoma formation rate of 6.8%, whereas Kanemaru et al. [4,21-23] reported no cases of tympanic cholesteatoma formation. Although Kanemaru et al. had a small number of cases, our study supports their findings as we also found no cases of tympanic cholesteatoma formation. However, as the number of cases accumulates, more cases of tympanic cholesteatoma may be encountered.

Limitations

As TMP closure surgery using gelatin sponge, recombinant bFGF, and fibrin glue had only been recently covered by the National Health Insurance, further long-term studies are necessary. It is necessary to find a way to achieve success in patients who have the habit of sniffing, which cannot be avoided through patient selection. Furthermore, it is necessary to be alert to long-term effects.

## Conclusions

Our results for 44 ears treated with gelatin sponge, recombinant bFGF, and fibrin glue showed high success rates for TMP closure and improvement of air-conduction thresholds and air-bone gaps. Factors contributing to the incomplete closure of TMPs were patient refusal of surgery more than once and the disappearance of the gelatin sponge one week after each surgery. The complications included two patients who had infections, but achieved successful closure; one patient who developed otitis media with effusion; and three patients who experienced transient vertigo due to local anesthesia. Our regenerative treatment for TMP demonstrated favorable results. Our novel technique is safe, simple, and less invasive than conventional myringoplasty.
